# Modeling the temporal network dynamics of neuronal cultures

**DOI:** 10.1371/journal.pcbi.1007834

**Published:** 2020-05-26

**Authors:** Jose Cadena, Ana Paula Sales, Doris Lam, Heather A. Enright, Elizabeth K. Wheeler, Nicholas O. Fischer

**Affiliations:** 1 Engineering Directorate, Lawrence Livermore National Laboratory, Livermore, California, United States of America; 2 Physical and Life Sciences Directorate, Lawrence Livermore National Laboratory, Livermore, California, United States of America; Oxford University, UNITED KINGDOM

## Abstract

Neurons form complex networks that evolve over multiple time scales. In order to thoroughly characterize these networks, time dependencies must be explicitly modeled. Here, we present a statistical model that captures both the underlying structural and temporal dynamics of neuronal networks. Our model combines the class of Stochastic Block Models for community formation with Gaussian processes to model changes in the community structure as a smooth function of time. We validate our model on synthetic data and demonstrate its utility on three different studies using *in vitro* cultures of dissociated neurons.

## Author summary

Neurons form complex networks that play a critical role in the development and aging of the brain, as well as in health and disease. Understanding how these networks form and evolve over time can lead us to advances in neuronal and cognitive health. Previous studies have mainly used summary statistics or graph features recorded at different points in time to analyze neuronal networks. However, this approach ignores the temporal dependency of these features and may lead to discovering spurious patterns in the data. In order to thoroughly characterize neuronal networks, time dependencies must be explicitly modeled. We present a statistical model that captures both the underlying structural and temporal dynamics of neuronal networks.

## Introduction

Neurons in the brain form incredibly complex networks with trillions of synapses that provide the basis for information processing and underlie all physiological functions of the brain [1]. These networks change and evolve over multiple time scales, and their temporal dynamics have been shown to play a critical role in development and aging [2], learning [3], as well as in health and disease [4]. Understanding how these networks adapt under different conditions, and how they can be actively manipulated, could have profound implications for advancing neuronal and cognitive health in the face of disease and aging.

Even though advances in non-invasive brain imaging techniques have enabled substantial advances in human brain studies, *in vivo* experimentation remains challenging. In this context, *in vitro* systems continue to play an essential role in the neuroscientist’s toolbox, providing a more controllable experimental framework to study the functional activity of neuronal networks. Dissociated neurons are commonly seeded and grown on multi-electrode arrays (MEAs), which are then used to record the electrical signals associated with neuronal firing. Thus, MEAs represent a means of non-invasively measuring the functional activity of neuronal networks that develop over time *in vitro*. Recently, MEAs have been leveraged to evaluate the effect of different compounds on neuronal activity [5, 6], to characterize functional differences in the activity of neurons from different brain regions (i.e., cortical and hippocampal) [7], and to investigate the impact of the extracellular matrix on neuronal activity [8]. However, observations from dissociated neuronal cultures do not always translate to animal models [9]. Therefore, in order for *in vitro* MEA systems to be truly used as bona fide proxies for *in vivo* system they need to be thoroughly characterized.

Electrophysiology studies with MEA systems are generally long-term studies monitoring neural network activity every few days over a period of weeks to months [7, 10]. Electrical signals produced by the cells in each recording session are represented as action potential spikes. The temporal organization of these spikes can be summarized into a number of features, which are subsequently used to compare recordings across time or experimental conditions. Traditional features include mean firing rate, inter-spike intervals, and number of bursts. These features are useful at summarizing the overall level of activity of the cultures; however, they fail to capture the complex network structures that neurons form on an MEA. To this end, more recent studies [10] have deployed graph-theoretical features, such as small-worldness, clustering coefficient, and density, to characterize neuronal cultures in MEAs. In either case, these features are analyzed at each time point (typically, referred to as *day-in-vitro* or DIV) independently, such that temporal dependencies are ignored.

In order to understand the temporal dynamics of *in vitro* neuronal networks under various experimental conditions, we present here a statistical model that explicitly captures both the underlying structural and temporal dynamics of neuronal networks on MEAs. Our model combines the class of Stochastic Block Models for community formation with Gaussian processes to model changes in the community structure as a *smooth* function of time. Inference is jointly performed on multiple graphs across recordings conducted over different time points. This allows us to learn 1) community structure, 2) how the community structure changes over time, and 3) how community structure varies across experimental conditions. We validate our model on synthetic data, and demonstrate its utility on modeling experimental data from three different studies wherein two or more types of cultures are compared.

### Functional network analysis in neuronal cultures

Graph theory provides a well-founded framework to study *in vitro* neuronal networks in MEAs. Typically, functional networks are derived from electrophysiology data by modeling electrodes as *nodes* of a graph and correlated spiking activity between electrodes as *edges* connecting nodes. It is known that *in vitro* functional networks exhibit important structural properties present in the corresponding *in vivo* networks. Neuronal cultures have *small-world* topology [10, 11], which is a common characteristic of complex graphs, such as social networks and the Internet. MEAs have shown that neuronal cultures develop *rich-club* topology [12], where a small group of tightly interconnected hub nodes is involved in exchange of information between separate areas of the network. This result mimics the earlier finding of rich-club structure on the human connectome [13]. By analyzing cultures on MEAs, it is possible to study changes in network structure under different experimental conditions in a controllable environment. For example, Srinivas et al. [11] found that the small-world structure is disrupted when cultures are exposed to glutamate to induce epilectic activity. Another line of research, and closest to our work, involves observing cultures across long periods of time—e.g., 30 days—to study how functional networks develop [7, 8, 10]. In these previous studies, the bulk of the analysis involves computing various graph-theoretic measures—i.e., clustering coefficient degree distribution, path length distribution, modularity—and comparing these measures across experimental conditions and time. In these studies, networks in each time point are treated independently. In the current work, we study the community structure of the networks and explicitly consider temporal relationships in our model. There are three primary motivations for this work. First, features measured in each DIV independently are prone to overfitting—i.e., finding patterns in each DIV that may be merely noise. Second, our proposed temporal model is learned on a larger dataset consisting of multiple DIVs, which reduces model uncertainty. Finally, our proposed model provides the opportunity to make predictions and estimate uncertainty on unseen future time points.

### Modeling community structure with the Stochastic Block Model

Even though graph measures provide a concise summary to compare networks across experimental conditions and time, these features by themselves are not sufficient to capture complex network structure and temporal evolution. Further, from a few observations, it is challenging to make general statements about the range of networks that can be observed in future experiments. These disadvantages motivate us to apply generative models, which try to capture the underlying statistical *rules* that produce the observed networks. In particular, we consider the Stochastic Block Model (SBM).

The Stochastic Block Model [14] is a generative model for community structure and graph formation. In the generative story for a single recording (i.e., a single device recorded at a particular DIV), each node *u* first chooses one of *k* groups or communities to join, which we denote by *z*_*u*_. Then, edges are generated based on the community assignment. The probability *η* that an edge (*u*, *v*) exists depends only on the group membership of *u* and *v*. Formally, let *G* be an unweighted undirected network of *n* nodes, *u* = {1, …, *n*}, and let $A\in R^{n\times n}$ be the adjacency matrix of *G*, such that *A*_*uv*_ = 1 if (*u*, *v*) ∈ *E* and 0 otherwise. Then, *A* is generated as follows:

$$\begin{array}{ccc} \pi & ∼Dirichlet(\alpha_{1},\ldots ,\alpha_{k}) & \\ z_{u}|\pi & ∼Categorical(\pi_{1},\ldots ,\pi_{k}) & \forall u\in \{1,\ldots ,n\}\\ \eta_{ij} & ∼Beta(a,b) & \forall i,j\in \{1,\ldots ,k\}\\ A_{uv}|\eta ,z & ∼Bernoulli(\eta_{z_{u}z_{v}}) & \forall u,v\in \{1,\ldots ,n\} \end{array}$$

If there are multiple networks—i.e., multiple MEA devices with cultures under the same experimental condition—we can extend the SBM to jointly learn the common community structure for all these networks. Such model would 1) reveal commonalities across cultures under the same condition, and 2) give us more information about the range of networks that are likely under a given experimental condition. In this model, we have a set of *d* networks with adjacency matrices *A*^(1)^, …, *A*^(*d*)^. All of these graphs have the same set of nodes—i.e., the same set of electrodes—whose communities are generated from the same base distribution with parameter *π*. Now, in the generative process, each node *u* chooses a different community in each device; the choice in device *d* is denoted by $z_{u}^{\left(d\right)}$. Then, we generate edges according a common distribution shared across devices. This model reflects our belief that all the cultures under the same experimental condition have the same underlying community generation process—i.e., same edge probabilities—but, because of the uncertainty of the seeding process and variations on the location of cells in the MEA, the particular community of a node may differ across devices. Formally, the model is the following:

$$\begin{array}{cc} \pi & ∼Dirichlet(\alpha_{1},\ldots ,\alpha_{k})\\ z_{u}^{\left(d\right)}|\pi & ∼Categorical(\pi_{1},\ldots ,\pi_{k})\\ \eta_{ij} & ∼Beta(a,b)\\ A_{uv}^{\left(d\right)}|\eta ,z^{\left(d\right)} & ∼Bernoulli(\eta_{z_{u}^{\left(d\right)}z_{v}^{\left(d\right)}}) \end{array}$$

**Fig 1:**
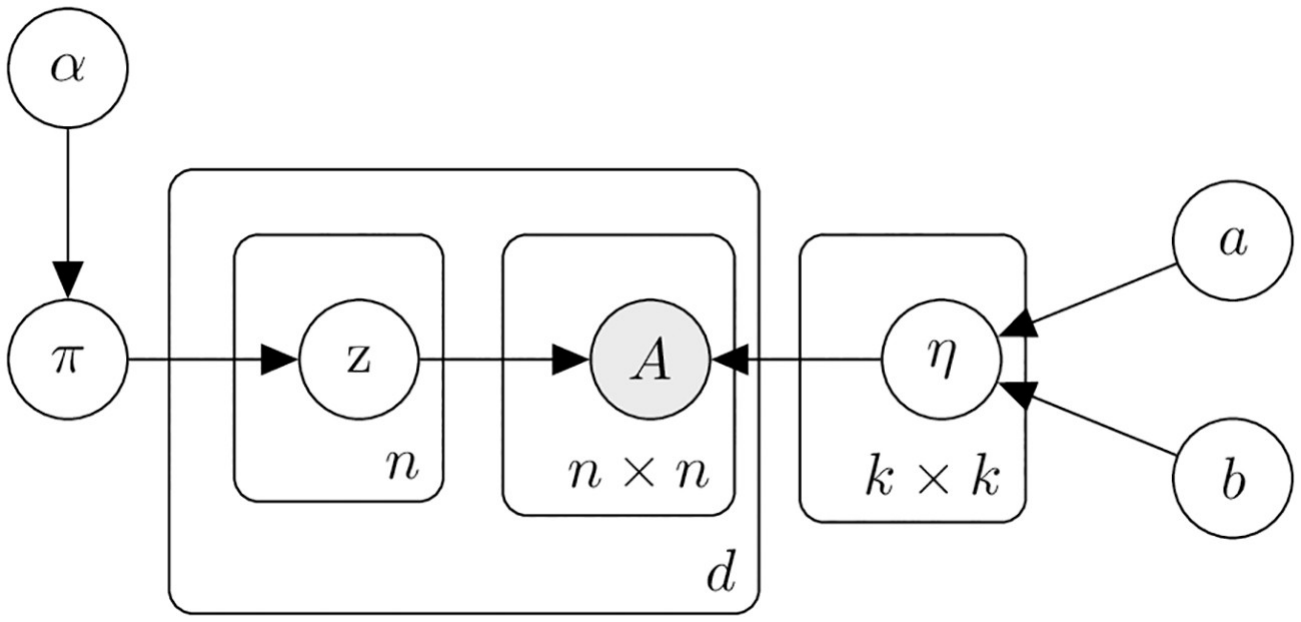
Plate diagram for the SBM on multiple networks for the same experimental condition. Each node *u* has a device-specific community assignment $z_{u}^{\left(d\right)}$. The edge probabilities, *η*_*ij*_, are shared across devices.

We show a plate diagram representation of the model in Fig 1.

## Materials and methods

### Temporal Stochastic Block Model (T-SBM)

We propose an extension of the SBM, called T-SBM, that performs inference on longitudinal network data, inferring the community structure of the graphs under study as well as the changes in the graphs over time. In the T-SBM we assume that the community assignment of each node is fixed across all time points but that the relationships across communities may change over time. An interpretation of this set of assumptions is that, once formed, the physical connections among neurons do not change. However, over time the strength—i.e., how much they are used— of the various connections change, resulting in different probabilities of edges within and between communities.

The primary impetus for the proposed model, over the standard static SBM, is to be able to model temporal dependencies of neuronal cultures as they develop and/or adapt to stimuli over time. In order to analyze longitudinal data with a standard SBM, one would need to fit a model to each DIV separately. It is not clear how to compare communities across time without some post-processing community disambiguation—which is a nontrivial problem. Such longitudinal study is straightforward with the T-SBM. Additionally, the temporal model allows us to make predictions and estimate uncertainty on unseen future time points as well as interpolation for DIVs where data was not recorded. Finally, because of constraints imposed by the Gaussian process as well as because of the fact that the temporal model is trained on more data, it is also more robust to noise than the static model and less prone to overfitting.

#### Model specification

We model the temporal dynamics of the neuronal networks with a latent function *x*(*t*) that varies smoothly across DIVs. Specifically, we impose a Gaussian process prior on *x* with squared exponential covariance structure:

$$\begin{array}{cc} \kappa (t,t^{\prime }) & =\sigma^{2}\;\text{exp}\;(-\frac{{\left(t-t^{\prime }\right)}^{2}}{2\ell^{2}})+\epsilon \\ x(t) & ∼GP(0,\kappa ), \end{array}$$

where *σ*, *ℓ*, and *ϵ* are parameters to be inferred. By applying a sigmoid transform, we convert *x*(*t*) into an edge probability *η*, which is a function of time.:

$$\begin{array}{c} \eta \left(t\right)=\text{sigmoid}\left(x\left(t\right)\right)=\frac{1}{1+e^{-x(t)}}. \end{array}$$

For ease of interpretation and to reduce the computational complexity of the model, we assume that the temporal changes can be modeled using two covariance functions, *κ*_in_ and *κ*_out_, corresponding to edge probabilities within and across communities, respectively. These covariance functions are used to generate latent functions *x*_*ij*_ for every pair of communities, which in turn are converted to edge probabilities by applying the sigmoid transform. That is,

$$\begin{array}{c} x_{ij}\left(t\right)∼\{\begin{array}{cc} GP(0,\kappa_{\text{in}}); & i=j\\ GP(0,\kappa_{\text{out}}); & i\neq j \end{array};\;\eta_{ij}\left(t\right)=\text{sigmoid}\left(x_{ij}\left(t\right)\right). \end{array}$$

**Fig 2:**
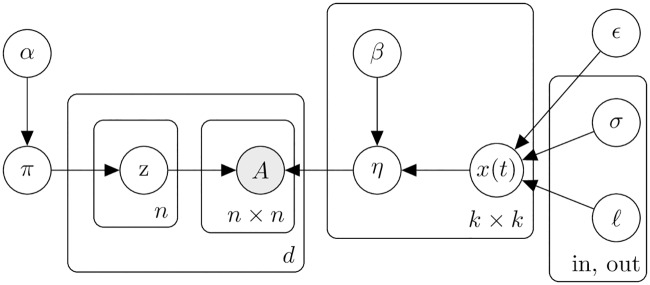
Plate diagram for the T-SBM. *η* is an time-evolving edge probability matrix governed by 1) a latent Gaussian process *x*(*t*) shared across community pairs and 2) a coefficient *β* that captures variations of specific community pairs.

Under this model, the temporal dynamics of the connectivity within all communities is governed by the same covariance structure, *κ*_in_, whose parameters will be jointly inferred from all the intra-community edges—analogously, for *κ*_out_ and inter-community edges. In order to also capture connectivity variations for each pair of communities, we let each latent process *x*_*ij*_ be a different sample of a Gaussian process, and we also infer an *offset* parameter *β*_*ij*_ for every pair of groups. Our proposed model, the T-SBM, is depicted in Fig 2 and is formally defined as follows:

$$\begin{array}{cc} \pi & ∼Dirichlet(\alpha_{1},\ldots ,\alpha_{k})\\ z_{u}^{\left(d\right)}|\pi & ∼Categorical(\pi_{1},\ldots ,\pi_{k})\\ \kappa_{\text{in}}\left(t,t^{\prime }\right) & =\sigma_{\text{in}}^{2}\;\text{exp}\;(-\frac{{\left(t-t^{\prime }\right)}^{2}}{2\ell_{\text{in}}^{2}})+\epsilon \\ \kappa_{\text{out}}\left(t,t^{\prime }\right) & =\sigma_{\text{out}}^{2}\;\text{exp}\;(-\frac{{\left(t-t^{\prime }\right)}^{2}}{2\ell_{\text{out}}^{2}})+\epsilon \\ x_{i=j}\left(t\right), & ∼GP(0,\kappa_{\text{in}})\\ x_{i\neq j}\left(t\right), & ∼GP(0,\kappa_{\text{out}})\\ \beta_{ij} & ∼N(0,1)\\ \eta_{ij}\left(t\right) & =\text{sigmoid}(\beta_{ij}+x_{ij}\left(t\right))\\ A_{uv}^{\left(d\right)}\left(t\right)|\eta ,z^{\left(d\right)} & ∼Bernoulli(\eta_{z_{u}^{\left(d\right)}z_{v}^{\left(d\right)}}\left(t\right)) \end{array}$$

### Multi-electrode array data

We considered three datasets collected from *in vitro* MEAs with 60 electrodes. In these studies, neurons were seeded and matured on MEAs whereby two or more experimental conditions were compared. We give a brief description of each dataset below and provide a summary in Table 1. Note that because the datasets were generated at different laboratories with differing experimental setups, we don’t combine data from different studies; rather, we analyze each one of datasets independently of the others.

**Table 1:** Datasets used in our experimental evaluation. In the “Experimental condition” column, the numbers in the parenthesis indicate the number of MEA devices for the corresponding experimental condition.

Name	Description	Experimental condition	DIV
Culture Complexity	Simple (primarily neurons) and complex (neurons, astrocytes, and oligodendrocytes) cultures	simple (5), complex (7)	11, 14, 18, 21, 25, 28, 31
Extracellular Matrix [8]	Neurons and glia grown with and without extracellular matrix (ECM) coatings	PDL (5), MaxGel (4), bECM (6)	13, 16, 20, 23, 27, 30
Neuronal Type [7]	Cortical (CTX) and hippocampal (HPC) neuronal cultures	CTX (5), HPC (2)	7, 11, 14, 18, 21, 25, 28

#### Culture Complexity

This experiment evaluated the functional differences between cultures containing primarily neuronal cells—with a small percentage of glial cells—and cultures where neurons and glial cells are grown together with cell ratios mimicking the *in vivo* environment (denoted here as *simple* and *complex* cultures, respectively). Cortical rat cells were grown on poly-D-lysine (PDL)-coated MEA devices, and 10-minute electrophysiology (ephys) recordings were taken at 3-4 day intervals over 31 DIVs.

#### Extracellular Matrix [8]

This study investigated the effect of culturing the neurons on MEAs with different extracellular matrix (ECM) coatings. Neurons co-cultured with glial cells were grown under three different ECM conditions: ECM extracted from decellularized brain tissue (bECM), a commercial ECM product (MaxGel), and control polymeric coating (PDL). 10-minute measurements were taken at 3-4 day intervals over 31 DIVs.

#### Neuronal Type [7]

This study assessed functional differences in cultures of mouse cortical (CTX) and hippocampal (HPC) neurons. 15-minute electrophysiology measurements were taken at 3-4 day intervals over 31 DIVs. The original data set had 32 CTX and 57 HPC devices. However, recordings were taken at different DIV, and most devices were missing one or more recordings in the duration of the study. In order to keep the evaluation consistent across all 3 studies, we only used those devices with recordings in all DIV considered in the study, resulting in 5 CTX and 4 HPC devices.

### Functional networks from electrophysiology data

**Fig 3:**
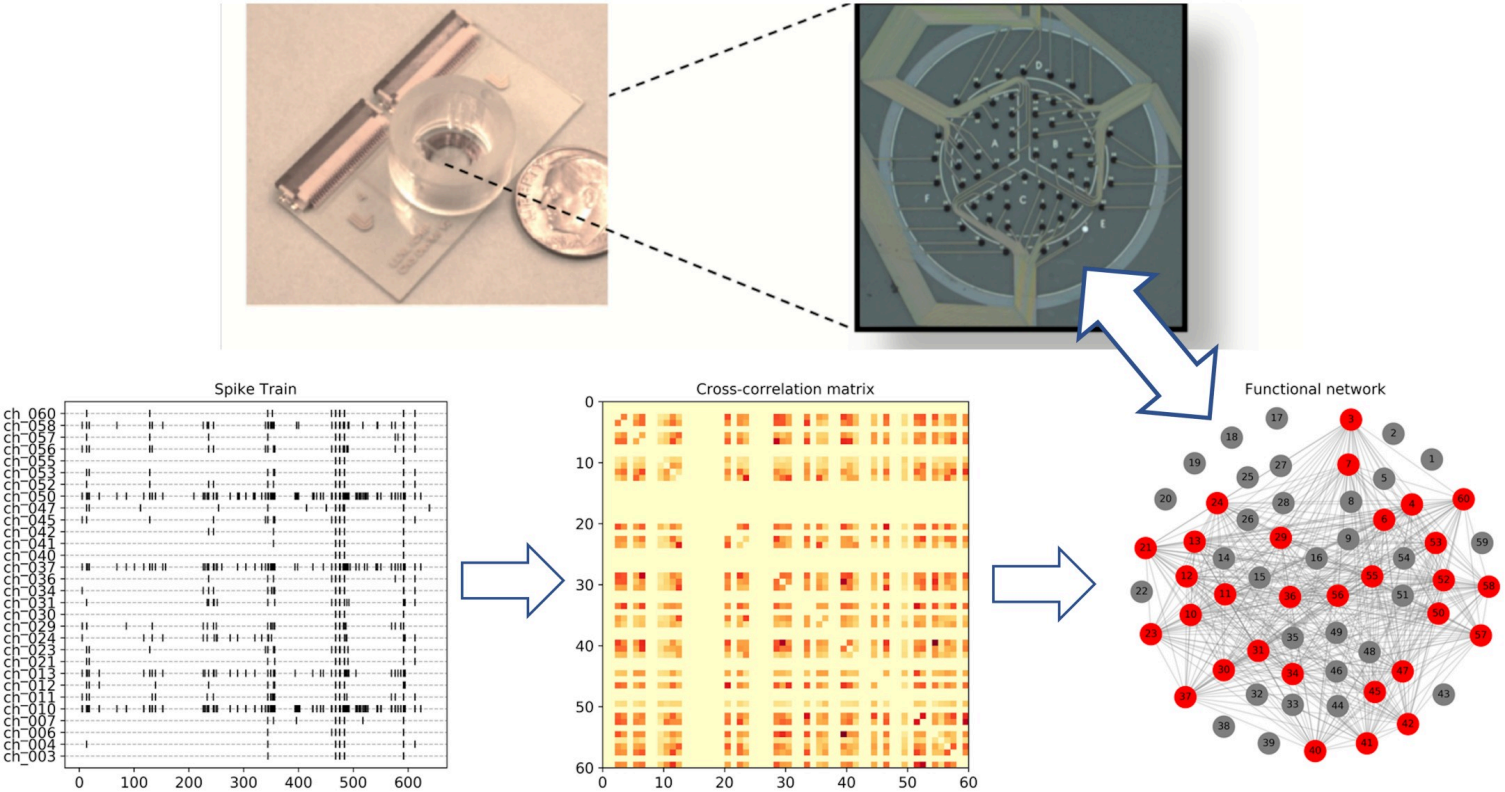
Modeling electrophysiology activity as a network. We compute the pairwise cross-correlation on active electrodes of a recording. Then, we construct a graph where the nodes are the 60 electrodes, and we add an edge between two electrodes if their cross-correlation is above some threshold.

We model an MEA as a graph where the set of nodes consists of the 60 electrodes in the device, and there is an edge between two electrodes if they show correlated activity. Because electrode activity is sparse (Fig 3 bottom left), directly measuring the cross-correlation of two electrodes on the entire recording time would yield artificially high correlation values and spurious edges. Instead, we followed a similar procedure to Downes et al. [10] for measuring correlation. First, we identified *channel bursts*, defined as 4 or more spikes in a channel occurring within a 100ms period, with the duration of the burst being the difference between the first spike and the last spike. Then, we defined *global bursts* as a time period when channel bursts start in 4 or more channels within 250ms of each other. Then, we measured the average cross-correlation over global bursts for every pair of electrodes, which gives us a cross-correlation matrix (Fig 3 bottom center).

Several methods of obtaining networks from correlation matrices have been previously explored. For instance, one could estimate the expected cross-correlation of two random spike trains and add an edge if the observed cross-correlation is significantly higher than this expected value [15]. Another possibility is to add edges in descending order of cross-correlation until the graph satisfies some property–e.g., until the graph is connected [16]. As noted by [17], all these methods have advantages and disadvantages; the most appropriate choice depends on the particular data set and goals of the study. We opted for the simpler approach of setting a fixed threshold and adding an edge between a pair of electrodes if their cross-correlation is above the threshold. In our experiments, we set this threshold to 0.20. As we discuss in the Results section, the choice of threshold affects all the networks similarly, so that it has little effect when performing a relative comparison of one network to another.

### Experimental setup

Inference was performed by Markov-Chain-Monte-Carlo (MCMC) sampling using the PyMC3 module for probabilistic programming [18]. We trained a separate model for each of the three datasets described above using all of the available devices and DIV. For the inference, we ran two MCMC chains with 5,000 samples each. We performed model selection for choosing *k*, the maximum number of communities, by varying it from 6 to 12 in increments of 2. We determined that *k* = 10 was a reasonable value; in all experimental conditions, the data was well-represented by less than 10 communities, so our choice of *k* did not arbitrarily constrain the model. We provide more details about choosing this parameter in S3 Appendix. We chose relatively “weak” hyperparameters for the prior distributions, which had little effect on the inference.

**Fig 4:**
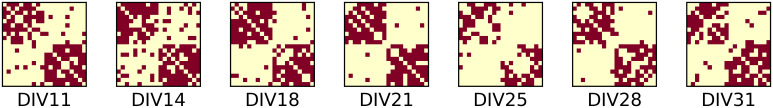
Adjacency matrices of synthetic networks generated for model validation.

For validation on synthetic data, we generated 4 networks of 20 nodes each. There were two communities in these graphs, each containing 10 nodes. The parameters for both covariance matrices were *σ* = .25, *ℓ* = 3, and *ϵ* = 0.01. For the *β* coefficients, we chose *β*_11_ = .5, *β*_12_ = −2, and *β*_22_ = .7. This configuration resulted in a *modular* network, with many edges inside the communities and only a few edges across. We show the adjacency matrices for temporal synthetic network generated with these parameters in Fig 4.

## Results

### Model validation using synthetic data

**Fig 5:**
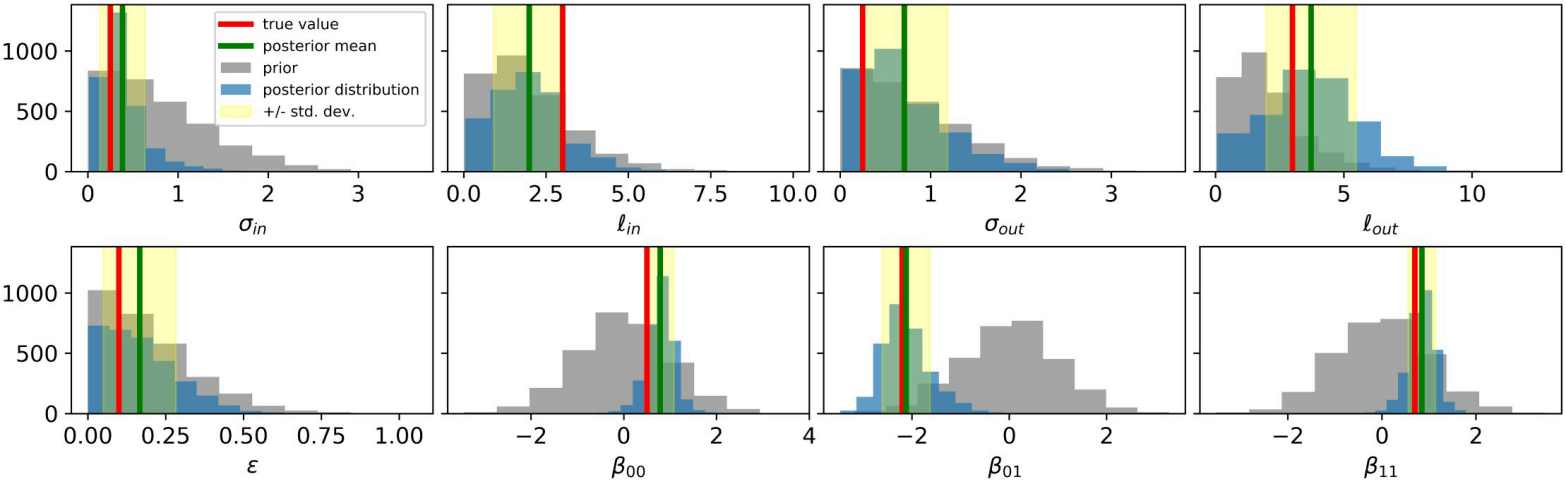
Inference on synthetic dataset. The T-SBM accurately recovers the true generative parameters even when the target notably differs from the prior distribution.

The T-SBM accurately inferred the true parameters in the synthetic data, namely the parameters of the covariance functions and the *β* coefficients associated with each pair of communities. Fig 5 summarizes the results of the inference. For each parameter, we show the prior and posterior distribution in gray and blue, respectively. We also show the posterior mean and the true value of the parameter—green and red lines, respectively. We observed that the posterior mean was very close to the ground truth for all parameters, even in cases where the true value had low probability in the prior distribution. We also analyzed the uncertainty of each estimate. The narrow standard deviation bounds on the *β* coefficients indicate that the model could infer these parameters with great precision, whereas there was more uncertainty on the estimate of the covariance.

We analyzed the inference of *z*, the community assignment vector. We took the last 500 samples of the MCMC trace and computed the adjusted mutual information score between the sample and true community assignment. We obtained an average score of 1, indicating that all the 500 posterior samples match the ground truth assignment.

### Application to functional network analysis on electrophysiology datasets

**Fig 6:**
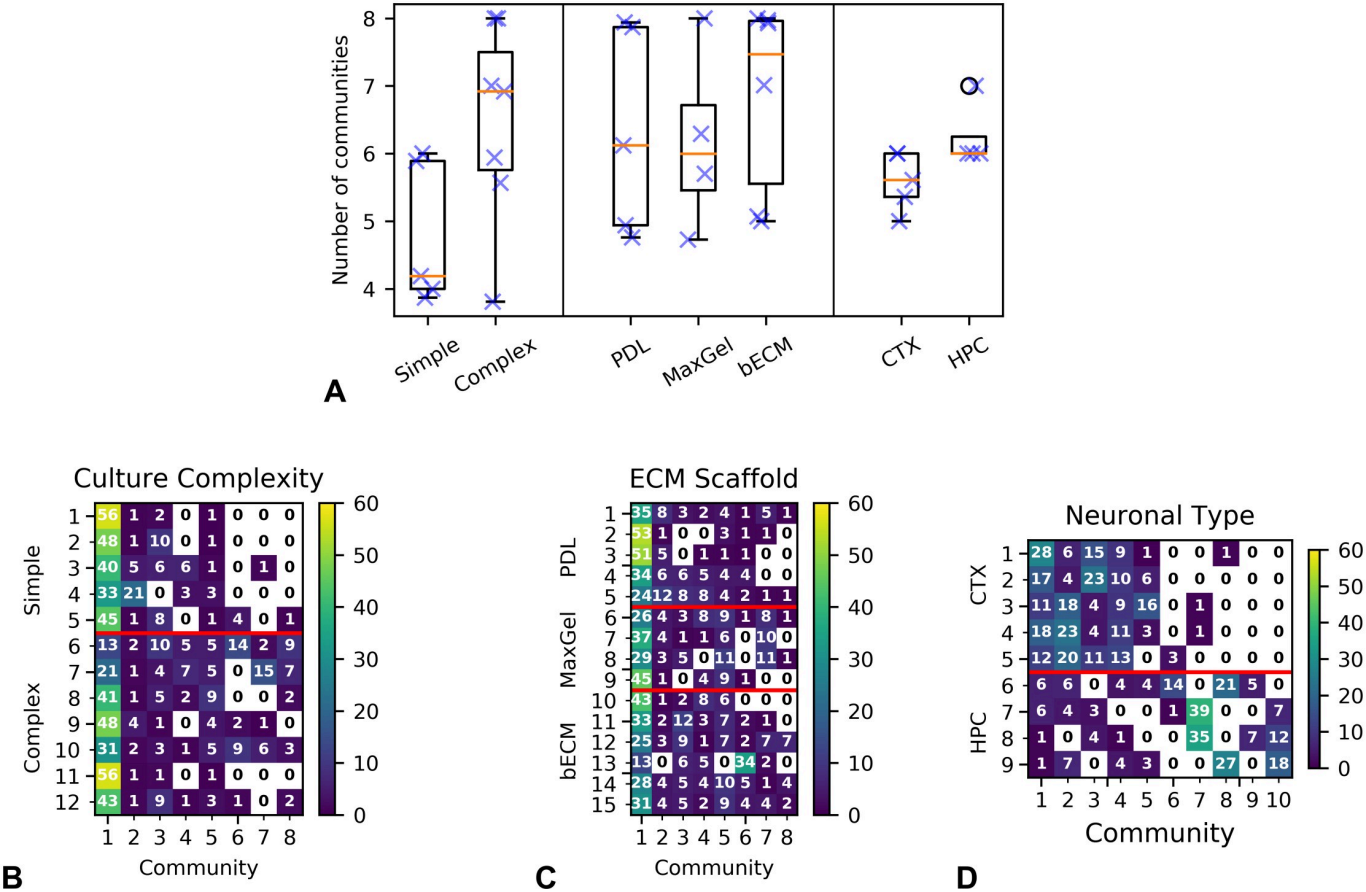
(A) Number of communities inferred by the model for the different experimental conditions—blue crosses correspond to individual MEAs. Community density in each device in the (B) Culture Complexity, (C) Extracellular Matrix, and (D) Neuronal Type datasets. White cells represent empty communities. We observed differences in community assignment when adding glial cells to simple cultures (B) or changing the cell type (D), but not from changing the ECM coating (C). See text for Discussion.

In Fig 6A, we show the inferred number of communities for each experimental condition. In most cases, the simple cultures (i.e., primarily neurons) developed less communities than the complex cultures, which contained glial cells at relevant ratios *in vivo*. In the Neuronal Type experiment, cortical networks formed fewer communities than hippocampal networks. It is noteworthy that, in this experiment, the variability in the number of communities was much smaller than the other two experiments. Finally, we did not observe significant differences across conditions in the Extracellular Matrix experiment, which is not entirely surprising given that the only difference across the cell cultures in this experiment was the ECM substrate coating, which was previously shown to accelerate neural network activity but not affect the structure of the cultures [8].

We then looked closely at how electrodes are distributed across communities. In Fig 6B–6D, we show the density of the communities in each device. Each row represents an individual MEA used in the study, and each column represents a community. Communities are ordered by size, so that community 1 is the most populated community. We note that community labels are consistent across rows—e.g., community 2 in row 3 is the same as community 2 in row 4. The numbers in each cell indicate how many nodes (i.e., electrodes) were assigned to a community in each device. Fig 6B reveals that both simple and complex devices shared a common core of communities, namely communities 1 through 5. However, in addition, electrodes in the complex networks occupied communities 6 through 8, meaning that these functional networks developed additional structures not observed in the simple devices. We observed a similar result in the Neuronal Type experiment (Fig 6D), where there was a noted separation between communities used in cortical and hippocampal networks. On the other hand, in the Extracellular Matrix experiment, there was little difference on community assignment across the three conditions (Fig 6C).

The discussion above is already useful to compare and contrast network structure in different kinds of devices. Additionally, the aggregate heatmaps from the previous figure provide one method for checking model parameters—e.g., ensuring that the maximum number of communities is not imposing unnecessary constraints on the model. Our next step is to examine the behavior of these communities and assign more descriptive labels. In particular, we focus on changes in connectivity over time for each experiment.

#### Culture Complexity

**Fig 7:**
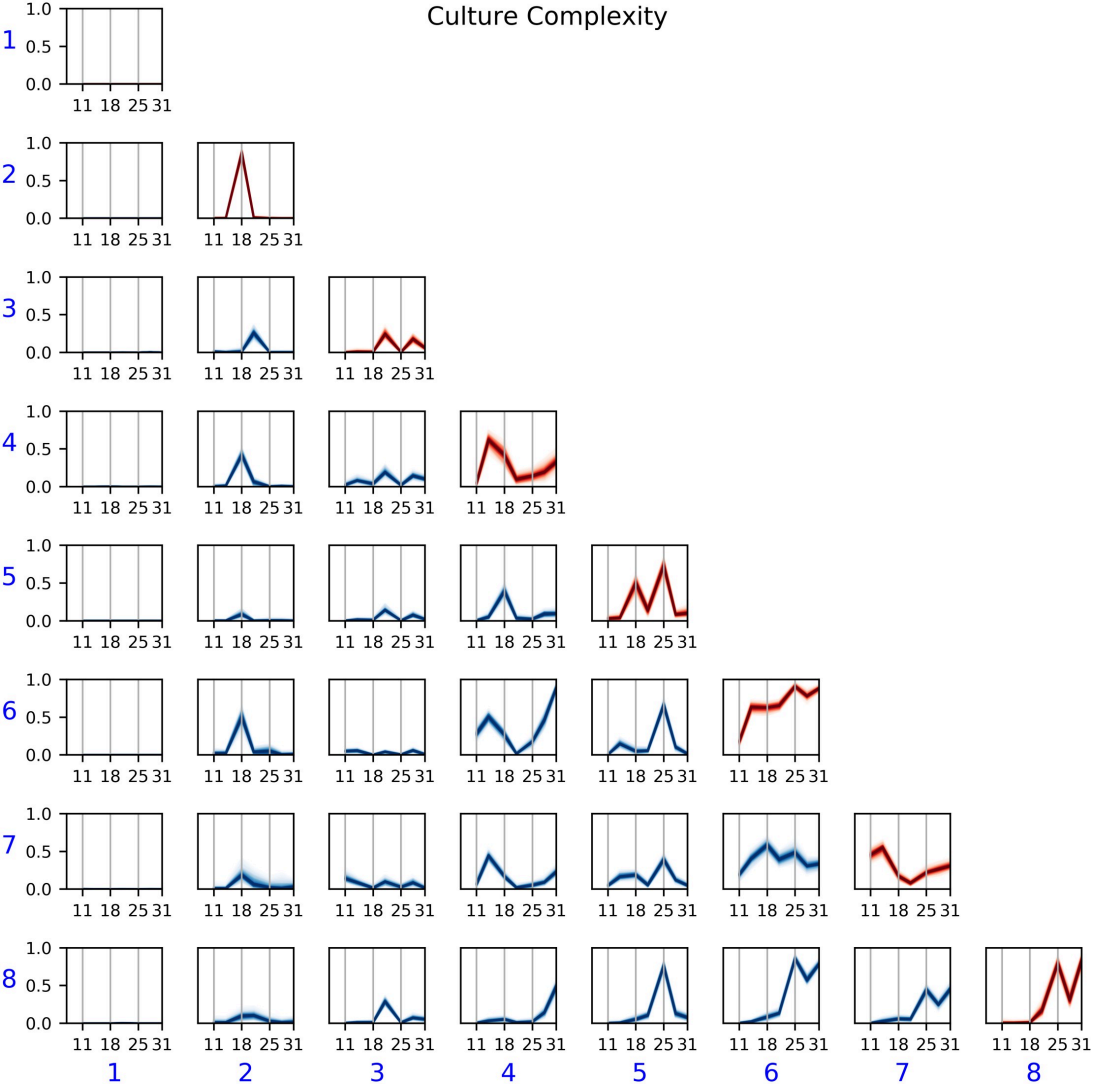
Posterior *η* as a function of DIV for the Culture Complexity experiment. Blue numbers denote community IDs, and a subplot in row *i* and column *j* shows the posterior *η*_*i*,*j*_—i.e., probability of connection—between a node in community *i* and a node in community *j*. Communities associated with complex devices (6–8) showed high probability of connection in later DIV.

In Fig 7, we show the posterior *η* parameters as a function of DIV. Recall that *η*_*ij*_ indicates the probability that a node from community *i* is connected to a node from community *j*. Since we modeled the MEAs as undirected networks, *η* is symmetric (i.e., *η*_*ij*_ = *η*_*ji*_), so we only show the lower half of the *η* matrix. A line plot in row *i* and column *j* shows the probability of connection *η*_*ij*_ (y-axis) across DIV (x-axis). We observed, for instance, that community 1 had connection probability close to 0 with every other community in all DIVs. Thus, community 1 denotes a community of *inactive* electrodes, those that never interacted with the rest of the device. As depicted in Fig 6B, most electrodes were assigned to the inactive community, either because they did not show any spike activity at all or because their activity was not correlated with that of other electrodes.

While easy to interpret, the behavior of community 1 is not particularly interesting, and it could be discovered without modeling by computing simpler summary features. A more interesting way to analyze Fig 7 is to focus on the communities associated with complex devices, namely communities 6, 7, and 8. A commonality of these communities is that they exhibited high connection probability in the later DIVs. For communities 6 and 8, the probability of an edge inside the community (*η*_66_ and *η*_88_) was close to 1 from DIV 25 onward. For community 7, *η*_77_ was relatively low; however, the probability of an edge from community 7 to communities 6 or 8 was at least 50% in the later DIV. These observations suggest that in this study:

1. Complex cultures are more likely to exhibit non-trivial network structure in later DIVs than primarily-neuron cultures.
2. Complex networks have one or two communities of synchronized electrodes—i.e., either 6 or 8, or both, are present—and a community that is only spuriously synchronized to the aforementioned one—i.e., 7.

Another way to use the model is to analyze the network structure of specific devices instead of making statements about an experimental condition as a whole. For example, in Fig 6B, we see that simple device 4 had 21 nodes in community 2, which is unusually high compared to all the other devices. Then, from Fig 7, this community had very high connectivity in DIV 18 and almost no connectivity elsewhere. Going back to the spike train for device 4 in DIV 18, we found a spike occurring simultaneously in all the electrodes assigned to community 2, explaining the behavior of the model.

#### Extracellular Matrix

**Fig 8:**
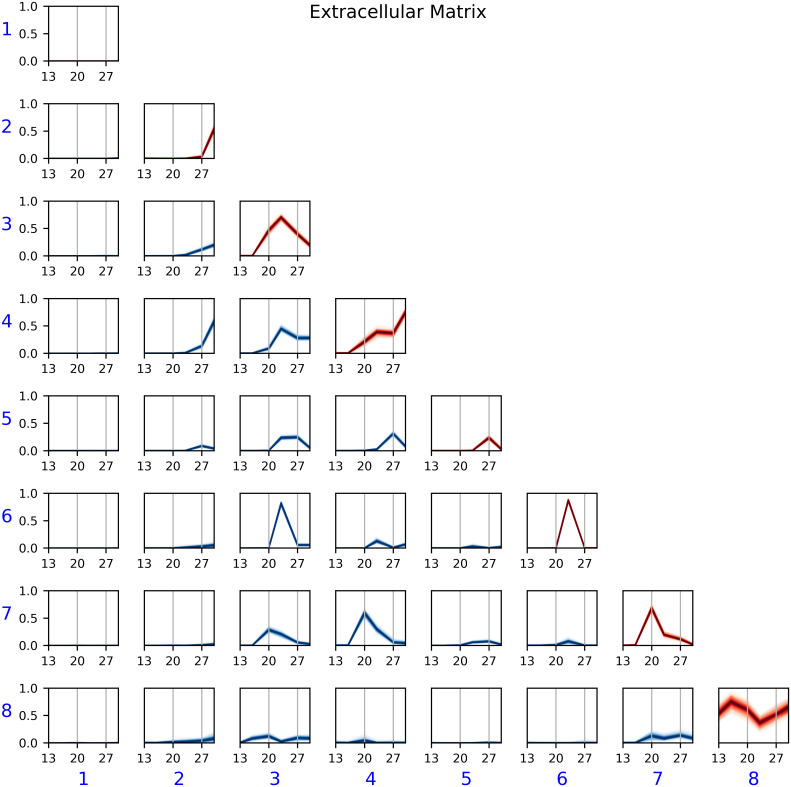
Posterior *η*(*t*) for the Extracellular Matrix experiment. The model captured various types of temporal behavior. Community 8—mostly associated with bECM cultures—showed high probability of connections throughout the duration of the study. Community 4, in contrast, showed an upward trend with time. The remaining communities captured *transient* behavior of electrodes that were correlated only in a particular DIV.

We performed a similar analysis for the Extracellular Matrix dataset. As with the previous dataset, Fig 8 shows that community 1 was the community of inactive electrodes. We also observed that, except for community 8, edge probabilities were close to 0 before DIV 20. Since community 8 was mostly present in bECM devices, the model agrees with the findings of [8] that bECM aids on early development of functional networks even though the three different ECM substrate coatings arrive at the same structure in later DIV. The other communities were generally present in all experimental conditions, revealing commonalities of the networks regardless of coating. Communities 2, 3, 5, 6, and 7 can be labeled as *transient*, since they showed heightened connection probability only during one DIV. Community 4 showed increasing probability over the duration of the study.

#### Neuronal Type

**Fig 9:**
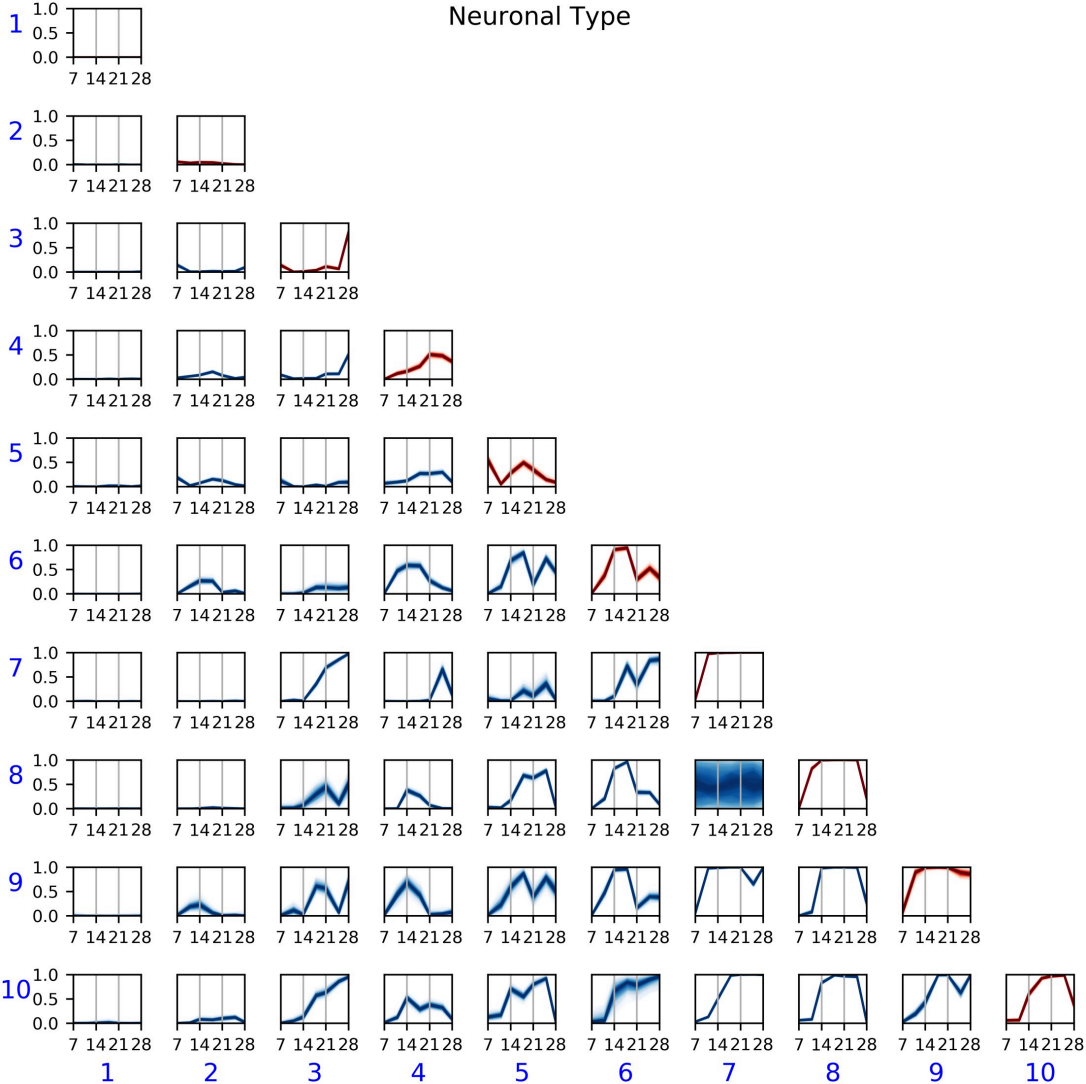
Posterior *η*(*t*) for Neuronal Type experiment. Communities 1–5, which were predominant in cortical cultures, showed similar temporal patterns to the cultures in the other studies above. Communities 6–10, associated with hippocampal cultures, formed almost fully-connected networks with high probability.

In this last study, we observed a more uniform distribution of nodes across communities for both cortical and hippocampal cultures. In contrast to the two previous datasets, in the current dataset, both CTX and HPC devices had fewer nodes allocated to the inactive community—community 1 in Figs 6D and 9. Recall from Fig 6D that communities 1 through 5 were mostly associated with cortical cultures, whereas communities 6 through 10 corresponded to hippocampal cultures. Examining the posterior *η* parameters, we observed that the former five communities had temporal trends comparable with the previous two studies; that is, we see an inactive community (1), transient communities (2, 3, and 5), and a community with increasing connectivity over time (4). Finding these similar trends is reasonable, since the two studies above were performed on cortical cells. In contrast, the communities in hippocampal networks were connected essentially with probability 1 for most of the duration of the study, showing a much higher level of synchronized activity than cortical cultures. Fig 9 also suggests that some of the communities in the hippocampal cultures were redundant, as they captured similar temporal trends. We could infer the same number of patterns—as well as increasing the interpretability—with a smaller value of the parameter *k* in the T-SBM.

### Effect of cross-correlation in graph modeling

**Fig 10:**
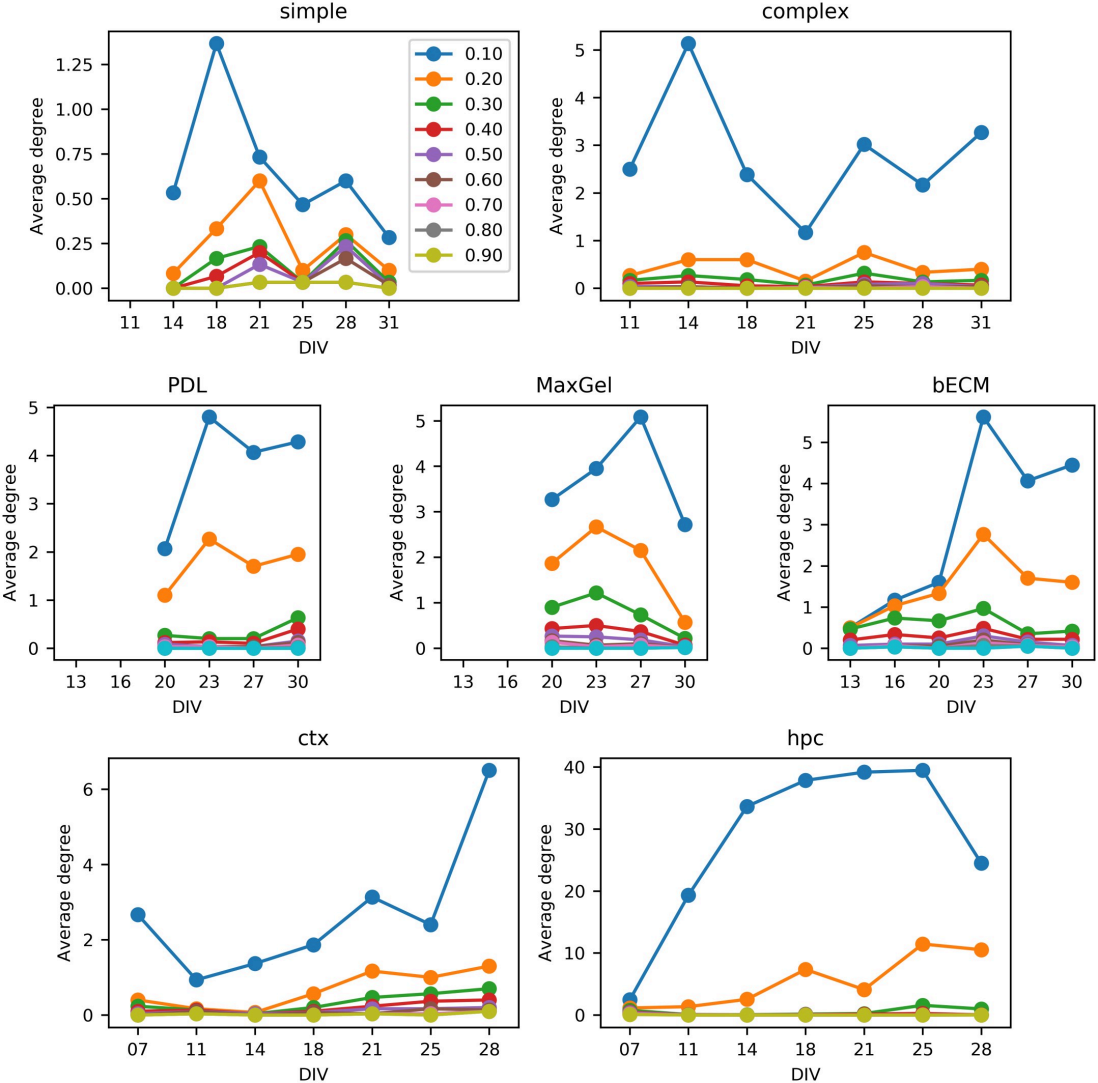
Average degree as a function of cross-correlation threshold for the Culture Complexity (top), Extracellular Matrix (middle), and Neuronal Type (bottom) datasets. Changes in the threshold do not affect the relative trend on graph properties, only the magnitude.

We analyzed the effect of the threshold applied to the cross-correlation matrix to obtain an unweighted network. In Fig 10, we show the average degree for the three datasets that we consider as we vary the threshold from 0.10 to 0.90. Increasing the threshold caused the average degree to decrease, which is reasonable, since the graphs had fewer edges. Importantly, this change was similar for all DIVs, and thus the threshold did not affect the relative trend of the graph property. We observed similar results for other graph features.

To further analyze the effect of the threshold, we repeated our experiments for the Culture Complexity dataset using values of 0.05, 0.10, and 0.30 cross-correlation. We did not examine higher thresholds because, as show in Fig 10, the graphs become almost completely disconnected at higher thresholds. We report these results in S1 Fig. Briefly, for thresholds 0.05 and 0.10, we observe the effects discussed in the Results section above, where the threshold was 0.20. That is, complex devices have more communities than simple devices, and these additional communities are associated with higher connectivity at later DIVs. For threshold 0.30, there are no noticeable differences in community structure across culture type, which is expected given the little connectivity.

**Fig 11:**
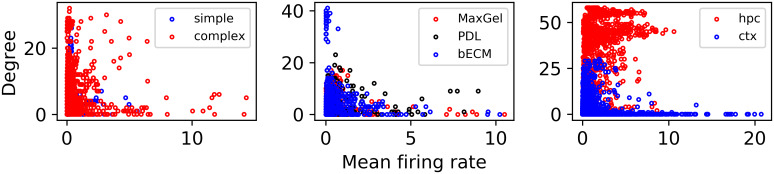
Mean firing rate compared to the degree of the nodes in our networks. Connectivity cannot be merely explained by the level of activity in an electrode.

We also studied the relationship between level of activity on the MEA and connectivity in our graph model. This analysis is useful to verify that increased graph connectivity is not merely a function of higher spike rates in the corresponding electrodes. In Fig 11, we compare the mean firing rate of each electrode in every device to its degree (i.e., number of connections) on the graph—each dot in the graph is one electrode of one MEA. We did not observe a correlation between firing rate and node degree, which indicates that the graph model provides additional information not captured by this simple spike train feature. As an aside, we also observed that, except for the Neuronal Type dataset, the mean firing rate by itself is not a useful feature for classifying experimental conditions.

## Discussion

We have presented a generative model for analyzing the community structure of *in vitro* neuronal cultures. Our applications to three experimental datasets show that the T-SBM has the modeling power to characterize different connectivity patterns encountered on *in vitro* networks, both in the same MEA and across time. By analyzing the parameters inferred using the T-SBM, it is possible to obtain a more in-depth characterization of the network dynamics that develop under different experimental conditions.

In the Results section, we discussed several observations obtained from the model in each dataset. These observations are in agreement with previous analysis of the data, but we also made some additional observations based on the temporal community modeling, which are missing from those previous studies. For instance, the T-SBM showed that hippocampal cultures tend to form more connected networks than cortical cultures, which has been observed before based on differences in correlation and interburst intervals [7]. In addition, we found that *all* communities in the hippocampal networks were highly connected throughout the duration of the study, whereas cortical networks contained both transient or sporadic communities, as well as communities that increased in connectivity over time. We also observed transient and sporadic communities in the Culture Complexity and Extracellular Matrix studies, which were conducted using cortical cells. Finally, analysis of the model also revealed some similarities and differences across the three datasets. In particular, we observed in Fig 6 that the communities appearing in cortical networks were different than those in hippocampal networks. In contrast, in the Culture Complexity study, the communities in complex cultures were a *superset* of those in the simple cultures. While further studies with larger datasets would be necessary for making definite conclusions about this, our results are consistent with previous studies on the functional networks of cortical and hippocampal cells [19, 20], where the networks from these two cell types are significantly different at least for some regimes. Our results on the other two datasets suggest that the addition of glial cells or extracellular matrix molecules to cortical neuronal cultures does not change the network structure; rather, it accelerates the formation of the networks [8].

### Modeling considerations and alternatives

There are various options to model temporal changes in the communities. We could assume that the community memberships of the nodes in the graph evolve over time, with nodes switching communities and communities merging together or splitting apart. In the model, this assumption corresponds to making $z_{u}^{\left(d\right)}$ a function of time. Alternatively, we could assume that the community assignment is fixed, but the connection probabilities, *η*, in these communities change with time. However, as noted by [21], allowing *both* the connectivity parameters and the group membership to vary simultaneously without any additional constraints leads to identifiability problems. In the T-SBM, we opted for modeling changes in *η* while keeping the group membership fixed over time. In the context of the generative process, this modeling choice amounts to assuming that each node chooses a community at the beginning of the process and never reconsiders this choice.

There are also several options for modeling the mechanism that drives the changes over time. One natural approach that has been previously proposed [21–23] is to make a Markovian assumption, such that the community memberships and/or connectivity at time *t* depend only in those at time *t* − 1. Then, as part of the inference, we would learn transition probabilities as well as the other parameters of the SBM. One drawback of these models, however, is that they implicitly assume that the observations are regularly-sampled in time—e.g., recordings are always taken in 3-day intervals—which is generally not the case with experimental MEA data. Instead, by using a Gaussian process prior, the T-SBM learns a continuous transition function, and it is resilient to irregular samples. Furthermore, the proposed model can interpolate missing DIVs to generate a distribution of plausible networks for the days where no measurements are taken.

We also note that other probabilistic models for temporal networks besides the SBM have been considered. Durante and Dunson [24] proposed a *latent space model*, where for each node *v* they learn a vector representation that varies over time, *x*_*v*_(*t*). Then, the probability that two nodes *u* and *v* are connected is proportional to the dot product of their vector representation. Similar to our setting, the temporal evolution (i.e., changes on the latent vectors) is modeled using Gaussian processes.

We emphasize that all the above modeling assumptions are reasonable, and the choice of model should be guided by domain knowledge as well as the goals of the study. For example, if we are interested in using a temporal model for predicting future network structure, we would prefer a model with higher predictive likelihood. In S2 Appendix, we compare our proposed T-SBM to the HMM-SBM of Matias and Miele [21] and to a static SBM for this predictive task. We trained the models with all data, except for the last DIV. Then, we compute the predictive likelihood for networks in the last DIV. The static SBM and the HMM-SBM have higher predictive likelihood in the simple devices. These devices have little connectivity across all DIVs, and the previous observation in time is a good predictor of the current observation. In the complex devices, T-SBM has generally better performance, but the static SBM is competitive. This observation suggests that our modeling assumption of fixed community structure over time is more appropriate for this dataset than the HMM-SBM, where nodes move around communities.

### Limitations and future work

As described in Materials and Methods, we defined functional networks based on cross-correlation between electrodes. As such, the results obtained from our model have to be interpreted in the context of cross-correlation as a measure of similarity and its inherent limitations. For example, if we discover a community with high intra-connection probability in all DIV, we can state that there is a group of electrodes all highly correlated to each other. However, it is not clear what is causing the high correlation (and thus the high connectivity)—i.e., one electrode communicating with others in a cascade vs. some external effect.

There are numerous directions of future work both in the modeling side and in applications. One promising extension is to increase the relevance of the networks by modeling the direction and strength of the edges. One way to do so is to consider the displacement that produces the highest cross-correlation between two electrodes, as well as the magnitude, instead of making the edge binary based on a fixed threshold. As for applications, probabilistic models like the one that we propose can be used to assess whether or not a chemical agent causes a significant change in the network. For example, in a study of epilepsy, we can train separately a T-SBM using a set of control cultures and a T-SBM for cultures exposed to an epilepsy-inducing chemical. Presumably, these two sets will have different community structures. Then, we could assess the efficacy of a treatment by applying it to an unhealthy culture and checking whether the community structure after the treatment is likely to be generated by the T-SBM trained on the control group.

### Conclusions

In conclusion, we demonstrate an approach to evaluate *in vitro* longitudinal network data, ensuring that the temporal dynamics are incorporated in an SBM framework. This approach inherently reduces model uncertainty while providing predictive capability for inferring future network function. We anticipate that this model will be used to provide greater understanding on the impact of exogenous effects on *in vitro* neuronal network function, including adverse effects from chemical, biological, and disease as well as restorative effects of therapeutics and countermeasures.

## Supporting information

S1 Appendix Consistency of inference across random initializations. (PDF)

S2 Appendix Comparison to HMM SBM for out-of-sample prediction. (PDF)

S3 Appendix Number of communities. (PDF)

S1 Fig Effect of threshold in the modeling results. (PDF)
