# Supplementary material for: Modeling the temporal network dynamics of neuronal cultures
Source: PLoS Comput Biol. 2020 May 26;16(5):e1007834. doi: 10.1371/journal.pcbi.1007834 (PMC7274455; doi:10.1371/journal.pcbi.1007834)
Supplement: S1 Appendix — (PDF) [file pcbi.1007834.s001.pdf]

S1 Appendix. Consistency of inference across random initializations

We ran the inference—i.e., MCMC chains—with 10 different random initializations for the Culture Complexity dataset. We then evaluated convergence across the chains according to different criteria.

In Fig A, we show the posterior distribution for the for the parameters of the Gaussian processes—that is, variances (scale) and lengthscales (l) σ^2_{in}, σ^2_{out}, ℓ_{in}, ℓ_{out}. We see that we arrive to similar posterior distributions for different initializations, with some variability for σ^2_{out}.

More formally, we computed the Gelman-Rubin ($\hat{R}$) diagnostic to evaluate whether all the chains arrive to the same posterior distribution. Values of $\hat{R}$ below 1.2 suggest that a collection of MCMC chains are converging to the same posterior distribution for some parameter. The test suggests convergence for the "in" parameters (i.e., $\hat{R} = 1.02$); however, the test suggests different posteriors for ℓ^2_{out} ($\hat{R} = 1.21$) and σ^2_{out} ($\hat{R} = 1.195$). On closer inspection, we noticed that one of the 10 chains contains samples with much lower likelihood than the other 9, which indicates that this chain has slower convergence or is "stuck" in some low-likelihood region (Fig B). If we remove this chain, the $\hat{R}$ values for ℓ^2_{out} and σ^2_{out} are 1.07 and 1.19, respectively, indicating convergence, albeit with high variability in σ^2_{out}. Because of consistency in these parameters, the results in Fig A are reliable across different initializations.

For the community structure, convergence diagnostics across chains is not an appropriate way to assess reliability, since community indexes have different meanings in different chains. For instance, community 1 in one chain may be equivalent to community 4 in a different chain. Instead, we evaluate the reliability of the community structure across multiple MCMC runs in terms of the adjusted mutual information score (AMI). Let z_{di} be the community for device d in the ith run. For each run i and device d, we have a collection of posterior MCMC samples of z_{di}. In order, to obtain a single representative community $\hat{z}_{di}$, we use the algorithm for consensus clustering of Ailon et al. [25]. $\hat{z}_{di}$ can be seen as the median community of the posterior samples. Then, we compute the AMI score of $\hat{z}_{di}$ and $\hat{z}_{dj}$ for every pair of chains i and j.

In Fig C, we show the AMI scores for each device in the Culture Complexity dataset. The distribution in each boxplot is over pairs of MCMC runs, so it is a distribution over $\binom{10}{2} = 45$ values. We observe that the AMI scores in each boxplot are always above 0.6, indicating a high degree of agreement in the communities across all chains and for all devices. Simple device 1 and complex device 6 have an AMI score of 1, meaning that the 10 MCMC runs discover the same community structure. These two devices show no activity in the entire study—i.e., they are graphs with no edges—thus, it should be trivial to find a consistent result across chains.

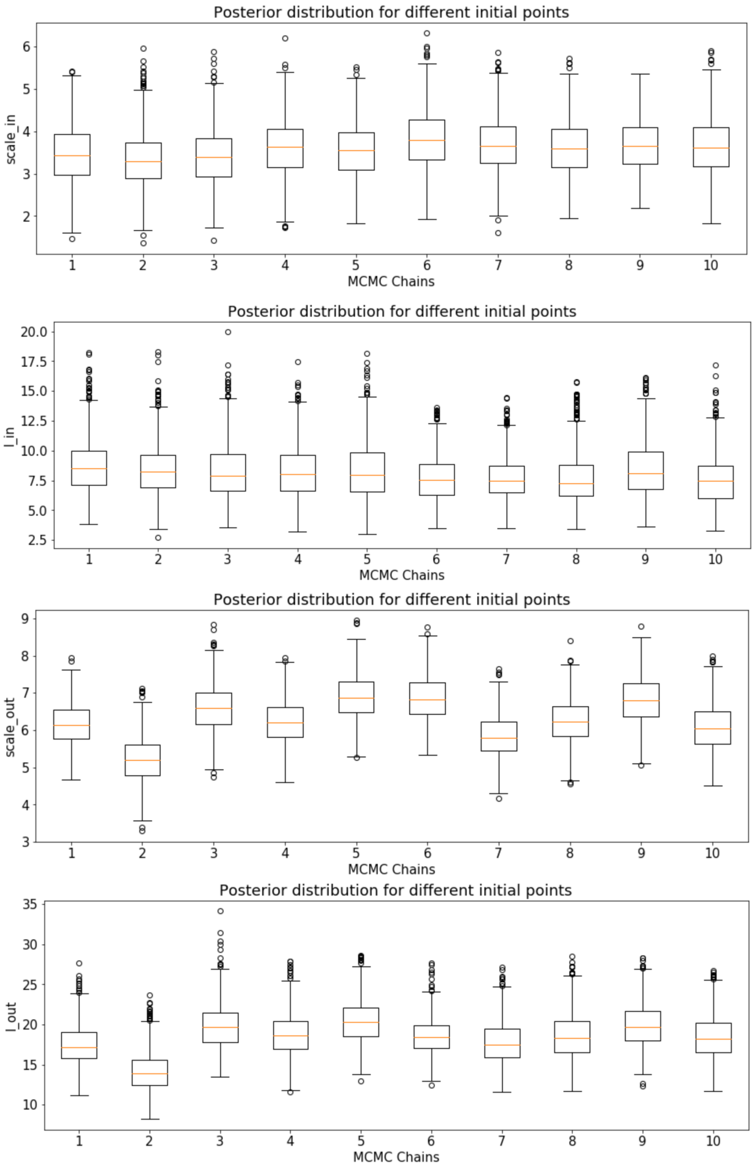

Fig A. Posterior distribution of the parameters of the Gaussian processes in the T-SBM for 10 random initializations. Results are consistent across different MCMC chains.

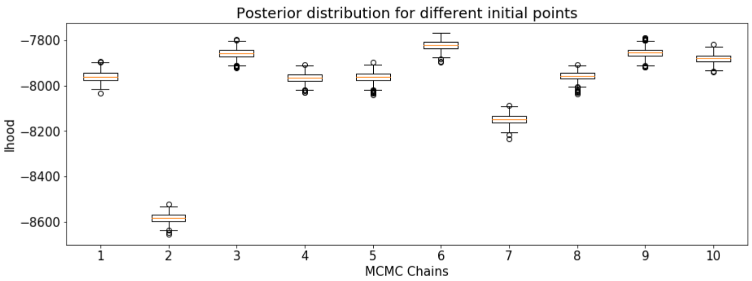

Fig B. Likelihood for 10 random initializations of the inference.

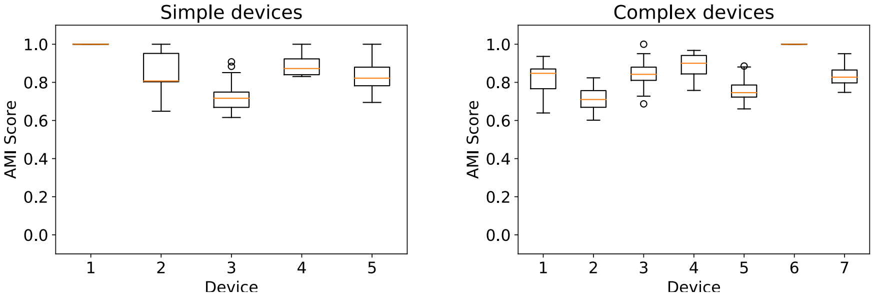

Fig C. Adjusted Mutual Information (AMI) score for communities obtained in 10 MCMC runs of the T-SBM. The AMI scores are above 0.6 in all cases, showing high agreement across MCMC runs.
