# Supplementary material for: Modeling the temporal network dynamics of neuronal cultures
Source: PLoS Comput Biol. 2020 May 26;16(5):e1007834. doi: 10.1371/journal.pcbi.1007834 (PMC7274455; doi:10.1371/journal.pcbi.1007834)
Supplement: S2 Appendix — (PDF) [file pcbi.1007834.s002.pdf]

S2 Appendix. Comparison to HMM SBM for out-of-sample prediction

We compared our proposed model to a static SBM and to the HMM SBM of Matias and Miele [21]. We used an R implementation of the model made publicly available by the authors. For the comparison, we trained all the models on the Culture Complexity dataset using data from DIVs 11 to 28. In the case of the static SBM, we only use data from DIV 28. The model of Matias and Miele makes a couple of subtle, but very relevant, different assumptions about the input data, namely:

- The HMM SBM implementation assumes that every node in the graph is *present* at least once among the time points. A node is said to be present at a given time point if it has at least one neighbor. We do not make this assumption in the results presented in our original manuscript, training our model with the full graph of 60 electrodes. For comparison purposes, we discard electrodes that do not satisfy this presence condition.

- The HMM SBM implementation takes as input the time series for a single graph (i.e., a single MEA), whereas our T-SBM implementation learns a model jointly for all the 12 MEAs of the Culture Complexity dataset. For the comparison, we fit a T-SBM to each MEA.

In order to make the comparison between the two models fair, we re-trained our model taking these assumptions into consideration.

For the evaluation, we computed the predictive likelihood for devices on DIV 31—the last time point of the data. In Table A, we show the predictive likelihood—in logarithmic scale, since the numbers are small—for each model and each device. There are 12 devices in the dataset, but one of the simple culture devices (S1) and one of the complex culture devices (C11) have no active electrodes for the duration of the study, so we remove them for evaluation, since they are trivial to model. The last column in the table indicates which model had the highest likelihood. We observe that both the static SBM and the HMM-SBM have better predictive likelihood on the simple devices. These correspond to networks with low connectivity, where the previously observed time point is a good predictor of the current time point. In the complex devices, T-SBM has generally better performance, but the static SBM is competitive. This observation suggests that our modeling assumption of fixed community structure over time is more appropriate for this dataset than the HMM-SBM, where nodes move around communities. The T-SBM has higher predictive likelihood for the entire dataset—i.e., sum of log likelihoods over all devices.

Table A. Predictive log-likelihood in the Complex Culture dataset. The T-SBM has higher likelihood overall. The static SBM and HMM-SBM have better performance in the simple culture devices.

**Device**	**Static SBM**	**HMM-SBM**	**T-SBM**	**Highest Likelihood**
S2	-24.076	-2.157	-6.804	HMM-SBM
S3	-8.407	-20.244	-29.111	Static SBM
S4	-0.560	-4.273	-9.575	Static SBM
S5	-16.208	-26.184	-19.274	Static SBM
C6	-479.686	-2102.622	-534.967	Static SBM
C7	-379.573	-521.861	-332.478	T-SBM
C8	-104.663	-188.184	-95.437	T-SBM
C9	-0.489	-1.564	-4.688	Static SBM
C10	-272.596	-371.689	-260.666	T-SBM
C12	-126.413	-154.938	-95.548	T-SBM
Total	-1412.670	-3393.716	-1388.548	T-SBM
