# Supplementary material for: Modeling the temporal network dynamics of neuronal cultures
Source: PLoS Comput Biol. 2020 May 26;16(5):e1007834. doi: 10.1371/journal.pcbi.1007834 (PMC7274455; doi:10.1371/journal.pcbi.1007834)
Supplement: S3 Appendix — (PDF) [file pcbi.1007834.s003.pdf]

S3 Appendix. Number of communities

When we state that we observed less than 10 communities, we mean that each device in each dataset used less than the 10 specified (Fig 6a in the manuscript). Using more than 10 communities would preserve this property at added computational cost and with the risk of overfitting. We note that the networks in these datasets have 60 nodes and some of these nodes are always disconnected from all others. For example, in the Culture Complexity dataset, in 10 out of the 12 devices, only 30 nodes or less have connections with others at any given DIV—the other two have more activity with 52 and 42 nodes showing connections. Given this small size, there is potential to overfit by adding more communities, especially because the number of η parameters grows quadratically with the number of communities.

In Fig D, we show the maximum number of communities used by a device in each dataset as a function of k, the maximum number of communities allowed. The dotted black line is plotted where the x-axis equals the y-axis for reference. We observe that the number of communities in a device grows with k. In the Culture Complexity and Extracellular Matrix datasets, the growth stops at 10 communities in a device, so increasing k for these devices would likely not reveal new structure. For the Neuronal Type dataset, we do not observe saturation even though individual devices are represented by a number of communities well below the maximum allowed. To further analyze the effect of higher k in this dataset, we show the community densities for increasing values of k in Fig E. For differnt values of this parameter, we observe a separation between communities used in cortical and hippocampal networks; one benefit of the extra communities is that this separation becomes more obvious, since the model has more flexibility.

Given 1) the small size of the networks, 2) the trends shown in Fig D, and 3) the consistent results for different k, we believe that modeling with 10 communities is appropriate to capture the structure of these datasets. We argue that one could set k anywhere in the range of 8 to 12 and find similar results. Values above will add to the computational cost and will likely not produce new insights, whereas values less than that would make it challenging to discern trends between different device types in each dataset we study.

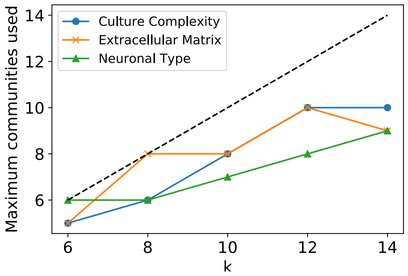

Fig D. Maximum number of communities used by a device in each dataset as a function of k, the maximum number of communities allowed.

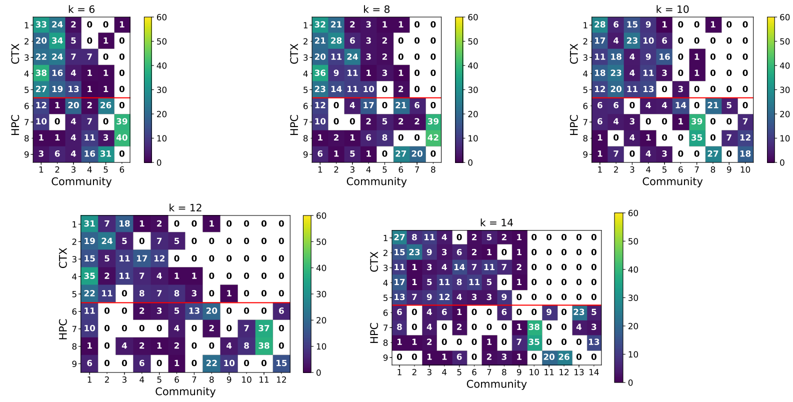

Fig E. Community density in the Neuronal Type dataset for different choices of k. We observe separation between the communities used in cortical and hippocampal cultures.
