# Supplementary material for: Modeling the temporal network dynamics of neuronal cultures
Source: PLoS Comput Biol. 2020 May 26;16(5):e1007834. doi: 10.1371/journal.pcbi.1007834 (PMC7274455; doi:10.1371/journal.pcbi.1007834)
Supplement: S1 Fig — (PDF) [file pcbi.1007834.s004.pdf]

S1 Fig. Effect of threshold in the modeling results 608

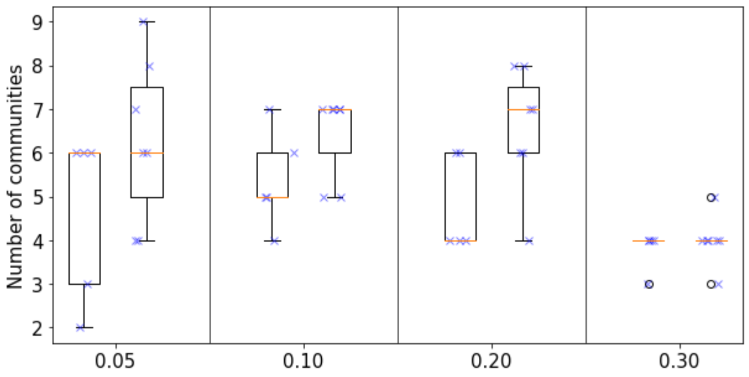

Fig F. Effect of cross-correlation threshold in the number of communities inferred by the T-SBM. We observe the same relative differences between simple and complex devices for a range of thresholds from 0.05 to 0.20. For 0.30 and possibly above, we observe little difference, since there is little connectivity in the graphs.
